# Knowledge, Attitudes, and Practices Associated with Human Papillomavirus Vaccine Recommendation Among Healthcare Professionals: A Cross-Sectional Study

**DOI:** 10.3390/idr17050126

**Published:** 2025-10-09

**Authors:** Layla M. Abdelhadi, Fatima S. Aryan, Rania Alsabi, Ghounan A. Samhan, Ayman M. Al-Qaaneh

**Affiliations:** 1 Nursing Faculty, Al-Balqa Applied University (BAU), Al-Salt 19117, Jordan; layla.abdelhadi@bau.edu.jo (L.M.A.); ghonan.samhan@bau.edu.jo (G.A.S.); 2 Princess Sarvath Community College, Al-Balqa’ Applied University (BAU), Amman 11931, Jordan; f.aryan@pscc.edu.jo; 3 Department of Community Health Nursing, Faculty of Nursing, Philadelphia University, Amman 19392, Jordan; raniaalsabi@gmail.com; 4 Faculty of Allied Medical Sciences, Al-Balqa Applied University (BAU), Al-Salt 19117, Jordan; 5 Department of Pharmaceutical Technology, Faculty of Pharmacy, Jordan University of Science and Technology (JUST), Irbid 22110, Jordan

**Keywords:** human papillomavirus vaccination, cervical cancer, healthcare professionals, knowledge, attitudes, practices, Jordan

## Abstract

Background: Cervical cancer remains a significant global public health concern, with human papillomavirus (HPV) vaccination serving as an effective preventive measure. Despite its proven efficacy, HPV vaccine uptake in Jordan remains low. This study aimed to assess the knowledge, attitudes, and practices (KAP) influencing HPV vaccine recommendation among healthcare professionals. Methods: A cross-sectional survey was conducted between August 2023 and February 2024 among 304 healthcare professionals and trainees in Amman, Jordan, using a pre-validated questionnaire. Descriptive statistics, correlational analyses, and Firth’s penalized logistic regression were employed to examine predictors of vaccine recommendation behavior. Results: Positive attitudes (OR = 3.89; *p* < 0.001) and active clinical practice (OR = 5.02; *p* < 0.001) were strong predictors of HPV vaccine recommendation. Unexpectedly, higher knowledge scores were associated with reduced likelihood of recommending the vaccine (OR = 0.44; *p* = 0.032). Significant variation in KAP scores was observed across professional groups, with physicians and academic staff demonstrating higher levels of engagement. Conclusions: Attitudes and practical engagement were more influential than knowledge alone in shaping HPV vaccine recommendation behavior among healthcare professionals. These findings underscore the need for interventions that not only enhance knowledge but also foster supportive attitudes and strengthen clinical advocacy skills. The results provide actionable evidence to inform targeted strategies for increasing HPV vaccine uptake and reducing cervical cancer incidence in Jordan.

## 1. Introduction

Cervical cancer remains a major global public health concern, ranking as the fourth most common malignancy among women. In 2020, an estimated 662,000 new cases and 350,000 deaths were reported worldwide, marking an increase from 604,127 cases and 341,831 deaths recorded in 2020 [1,2,3]. Persistent infection with high-risk human papillomavirus (HPV) types is recognized as the primary etiological factor in the development of cervical cancer, accounting for the vast majority of cases [1,4]. Globally, HPV16 and HPV18 are implicated in 70% of cervical cancers, contribute substantially to other anogenital and oropharyngeal malignancies [4,5,6,7]. Although most HPV infections resolve spontaneously within two years, a subset persists and can progress to malignancy, with persistent infection responsible for 95% of cervical cancer cases [1,3,4]. While many HPV types are benign, certain oncogenic strains are directly associated with carcinogenesis in women [1,6].

HPV prevalence varies considerably by region. A 2025 systematic review estimated that among women aged ≥50 years with normal cytology, the pooled prevalence of any HPV infection remained substantial, with high-risk HPV detected in 43% of cases [8]. These findings underscore the persistence of HPV infection in older age groups, highlighting the long-term public health burden. In Western Asia, approximately 2.5% of women harbor HPV-16/18 infection at any given time, and 72.4% of invasive cervical cancers in this region are attributable to these two genotypes [9]. In Jordan, a study conducted among women attending the Gynecology Clinic at Prince Hamza Hospital reported a 4% HPV infection rate [10]. National estimates indicate that approximately 115 women are diagnosed with cervical cancer annually in Jordan, with 71 deaths attributed to the disease. The age-standardized mortality rate of cervical cancer associated with HPV is estimated at 1.9 per 100,000 women [9,10]. The safety and efficacy of HPV vaccines are well established, with clinical trials and post-licensure surveillance consistently demonstrating strong protection against HPV16/18 and other high-risk types [11,12]. The World Health Organization (WHO) updated its guidance in 2022, endorsing a single-dose schedule as sufficient for individuals aged 9–20 years, with two doses for those aged 21–30 years and three doses for immunocompromised persons [2]. The U.S. Centers for Disease Control and Prevention (CDC) recommends routine vaccination at ages 11–12 (starting as early as 9), with a two-dose schedule for those initiating before age 15 and a three-dose schedule for those aged ≥15 years or immunocompromised [5]. Both WHO and CDC also recommend extending HPV vaccination to boys and men, recognizing the growing burden of oropharyngeal and other HPV-associated cancers among males [2,5].

Despite these advances, global disparities in vaccine coverage persist and are frequently shaped by healthcare professionals’ (HCPs) knowledge, attitudes, and clinical practices [12]. Jordan has not yet introduced HPV vaccination into its National Immunization Program, leaving access limited to private-sector purchase and creating significant cost barriers [9]. Local studies underscore these challenges: among female health sciences students, only 3.6% reported prior vaccination, though willingness rose to 75% if the vaccine were free [13]. A 2024 national study by Al-Leimon et al. further confirmed widespread knowledge gaps and vaccine hesitancy among the Jordanian public, while emphasizing that improved education and accessible testing strategies could significantly enhance uptake [14]. Surveys of pharmacists and medical students also confirm persistent knowledge gaps and mixed attitudes, despite broad support for eventual program introduction [15,16]. Earlier work among obstetricians and gynecologists similarly identified limited awareness and inconsistent recommendation practices [17].

Barriers such as cultural misconceptions, vaccine hesitancy, and insufficient training continue to hinder vaccine uptake. In Armenia, some physicians voiced concerns that HPV vaccination may promote early sexual activity [18], while in France, gaps in knowledge among school staff, particularly regarding vaccine safety and efficacy, were documented [19]. Despite the vaccine’s well-established effectiveness in preventing cervical cancer, uptake remains suboptimal in many regions [20]. International experiences further highlight the importance of provider engagement. In Serbia, medical students who received HPV education were more likely to recommend vaccination [21], while in Qatar, physicians’ willingness to vaccinate their own daughters correlated with stronger advocacy [22].

As frontline advocates, HCPs are instrumental in improving vaccination rates. In Jordan, although several studies have examined HPV awareness and vaccine acceptance among students, parents, pharmacists, and gynecologists [13,14,15,16,22], there is still no comprehensive investigation into the knowledge, attitudes, and practices of healthcare professionals themselves. This gap is critical, as HCPs play a pivotal role in shaping public trust, counseling families, and recommending vaccination. Addressing this gap is essential to inform future national strategies, guide targeted education, and support the integration of HPV vaccination into public health policy. Therefore, this study aims to assess the knowledge, attitudes, and practices associated with HPV vaccine recommendation among healthcare professionals in Jordan, with the goal of identifying gaps that may inform future policy and targeted educational interventions.

## 2. Material and Method

### 2.1. Study Design and Participants

This cross-sectional study was conducted using a structured, self-administered online questionnaire administered via Google Forms to assess HCPs’ knowledge, attitudes, and practices (KAP) regarding HPV vaccination. The study population included licensed HCPs and trainees residing in Amman, Jordan, comprising physicians, nurses, midwives, pharmacists, and laboratory clinicians, as well as academic staff from health-related faculties and senior health sciences students who are future HCPs. Participants were recruited from diverse healthcare settings, including public and private hospitals, outpatient clinics, and primary health centers, and university networks. The survey link was disseminated via institutional channels and professional groups. Inclusion criteria required participants to be at least 18 years of age, hold active professional licensure, and be currently employed in a clinical role, or be engaged in academic or clinical training within the geographical boundaries of Amman Municipality. Exclusion criteria included non-health-related administrative staff, individuals working outside Amman, and survey submissions with less than 80% item completion, which were excluded from analysis.

### 2.2. Study Instrument

The survey instrument was a 60-item questionnaire in English, originally developed and validated by Nguyen et al. [23], used with the author’s permission and adapted for the Jordanian context. Adaptations included minor linguistic modifications, culturally relevant phrasing, and references to national healthcare services. The questionnaire was based on the Theory of Planned Behavior (TPB) [24], and was linguistically and culturally adapted for use in Jordan to enhance contextual relevance:

1. The knowledge domain comprised 10 items (Items 14–23) assessing awareness and understanding of cervical cancer, HPV infection, prevention, and screening procedures. In the TPB, knowledge is not a core construct, but it is often included in health behavior studies as a background variable. It supports the formation of attitudes, shapes perceived control, and enables informed behavioral intentions.
2. The attitude domain included 9 items (Items 24–32), reflecting the attitudes toward HPV vaccination (TPB construct 1), including perceived safety, effectiveness, and necessity of the vaccine.
3. The practice domain, corresponding to the combination of subjective norms, perceived behavioral control, and behavioral intention under the KAP model, contained 23 items (Items 24–46):
  - The subjective norms domain, aligned with TPB construct 2, was captured by 5 items (Items 33–37) that explored perceived peer and institutional expectations regarding HPV vaccine recommendation.
  - The perceived behavioral control domain (TPB construct 3) included 6 items (Items 38–43) assessing healthcare professionals’ self-efficacy and perceived barriers in vaccine advocacy and delivery.
  - The behavioral intention domain (TPB construct 4) was measured by 3 items (Items 44–46) that evaluated the respondent’s likelihood of recommending or administering the HPV vaccine in the future.

The Knowledge section had categorical responses (Yes/No/Unsure or True/False/Unsure). Responses were dichotomized by coding correct answers as 1 and incorrect/unsure responses as 0. The knowledge score was then calculated as the mean score across items, yielding a continuous score ranging from 0 to 1. Attitude and practice sections utilized 5-point Likert scales, and domain scores were calculated as the average of items within each domain.

To ensure data quality, incomplete submissions (<80% of items answered) and duplicate responses (based on IP address and timestamp) were checked to be excluded from the analysis. The survey remained open for six months, with four reminder notifications sent to encourage participation. The survey was pilot tested among a small group of 20 participants to ensure validity, reliability, and comprehension before wider dissemination.

The questionnaire was prefaced by a page explaining the nature and objectives of the study and the voluntary nature of participation with a consent statement if they would like to take part in the study. The participants were assured that the outcomes of the research would not be used for routine appraisal of the participants. The questionnaire comprised sections on demographics (age, gender, profession, years of education), clinical experience (work setting and institution type), and KAP related to HPV vaccination. Participation was voluntary; informed consent was obtained electronically at the beginning of the survey, and responses were self-reported and anonymized at the point of entry.

Although the original instrument had undergone prior validation, formal content validity procedures were not repeated in this study. However, the adapted instrument was reviewed in face validity by three local experts in public health and Gynecology to ensure contextual relevance and clarity for the Jordanian healthcare settings. A pilot test was conducted with 20 HCPs to ensure clarity and contextual relevance. The instrument demonstrated excellent internal consistency (Cronbach’s α = 0.92) and high test–retest reliability (r = 0.89).

### 2.3. Data Collection

Data were collected from August 2024 to February 2025. Trained research assistants electronically distributed the survey link to 500 eligible participants through institutional email lists, professional networks, and social media groups. The online questionnaire, developed in Google Forms, was adapted from previous validated KAP surveys on HPV vaccination. Of the distributed questionnaires, 304 were completed and met the inclusion criteria (80% item completion, no duplicate responses based on IP address and timestamp), resulting in a response rate of 60.8%.

### 2.4. Sample Size Calculation

Sample size was calculated using G*Power 3.1 [25] based on Firth’s penalized logistic regression, selected as the primary analysis method due to potential rare-event distribution in vaccine recommendation patterns. Assuming an odds ratio of 2.0, 80% power, α = 0.05, and four predictors, a minimum sample size of 299 was determined. A total of 304 complete responses were collected, exceeding the calculated requirement, and yielding a response rate of 100%. Firth’s method was chosen over standard logistic regression due to its suitability for small-to-moderate samples, its robustness against complete separation in categorical predictors, and its superior performance with imbalanced outcome distributions. Analysis followed current methodological standards, including use of profile likelihood confidence intervals and convergence diagnostics.

### 2.5. Statistical Analysis

All analyses were conducted using IBM SPSS Statistics (Version 30) [26]. Descriptive statistics were used to summarize participant characteristics and KAP scores, with categorical variables expressed as frequencies and percentages, and continuous variables presented as means ± standard deviations or medians with interquartile ranges, depending on distribution. Nonparametric tests were used due to non-normality of KAP scores (Shapiro–Wilk *p* < 0.001). The Kruskal–Wallis test served as the omnibus test for comparing scores across professional subgroups. Post hoc pairwise comparisons were conducted using Dunn’s test with Bonferroni correction to control the family-wise error rate across 21 comparisons per domain. Rank-biserial correlation coefficients (r) were reported as effect sizes, interpreted as small (0.1), medium (0.3), or large (0.5).

Associations between categorical variables (e.g., profession and training status) were assessed using Pearson’s χ^2^ tests with Yates’ continuity correction for 2 × 2 tables. Fisher’s exact test was applied when expected cell counts were <5. Effect sizes were reported using Cramer’s V for nominal variables and gamma coefficients for ordinal relationships. Firth’s penalized logistic regression was used to identify predictors of HPV vaccine recommendation (agree vs. disagree). Independent variables included continuous KAP scores and categorical demographic/professional factors. Odds ratios (ORs) with 95% confidence intervals (CIs) were reported. All statistical tests were two-tailed, with significance set at α = 0.05. Epsilon-squared (ε^2^) values were reported for Kruskal–Wallis tests. Missing data (<5%) were managed using pairwise deletion after confirming randomness via Little’s MCAR test (*p* = 0.12).

### 2.6. Ethical Considerations

The study was approved by the Institutional Review Board (IRB) of Al-Balqa Applied University (approval number: 2024/2023/7/69). Electronic informed consent was obtained from all participants prior to survey completion. Data were collected anonymously, and no personally identifiable information was recorded to ensure participant confidentiality.

## 3. Results

### 3.1. Socio-Demographic Characteristics

A total of 304 healthcare professionals participated in this cross-sectional study conducted across hospitals and healthcare centers in Amman, Jordan (Table 1). The sample reflected a predominantly early-career workforce, with 32.2% (*n* = 98) aged between 30 and 39 years and 56.3% (*n* = 171) reporting five years or less of professional experience. The gender distribution was skewed toward females, comprising 79.3% (*n* = 241) of the total sample, consistent with national trends in the Jordanian healthcare sector. Regarding religious affiliation, the majority of participants identified as Muslim (95.7%, *n* = 291), reflecting the broader demographic composition of the country. Professionally, the cohort included nurses (44.1%, *n* = 134), midwives (22.4%, *n* = 68), physicians (17.1%, *n* = 52), and pharmacists (9.9%, *n* = 30). The remaining 6.5% (*n* = 20) comprised dentists, academic faculty, and clinical technicians. Most respondents were employed in urban public healthcare institutions (63.2%, *n* = 192), followed by those working in private-sector facilities (33.9%, *n* = 103) and military healthcare settings (3.0%, *n* = 9).

### 3.2. Descriptive Statistics and Interprofessional Variation in KAP Scores

#### 3.2.1. Knowledge

**Figure 1:**
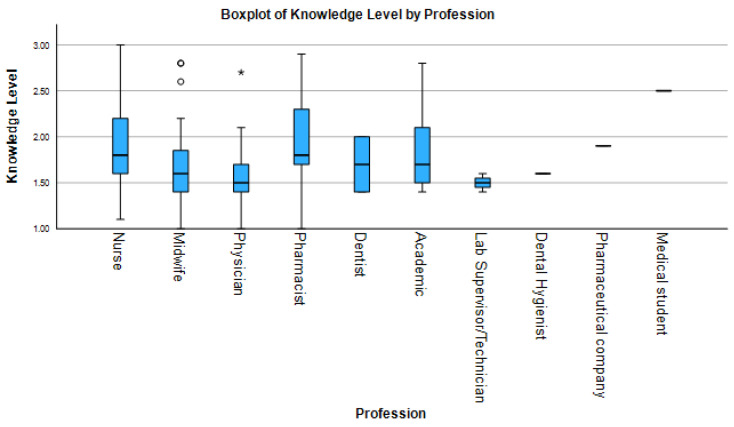
Boxplot comparisons of HPV-related knowledge scores across healthcare professions. Center lines show medians, boxes represent interquartile ranges (IQR), and whiskers extend to 1.5× IQR. Individual points denote outliers. Brackets show significant Dunn’s test comparisons with effect sizes.

Knowledge of HPV vaccination varied significantly across professional groups (Figure 1). Medical students demonstrated the highest knowledge levels, with a median score of 88 (IQR 80–94). Pharmaceutical professionals also showed high knowledge, with a median score of 70 (IQR 62–76). Academics (median 80, IQR 75–88) and pharmacists also scored relatively high, though with greater variability. In contrast, laboratory technicians (data not provided in table) showed uniformly low knowledge scores. Nurses (median 72, IQR 65–80), midwives (median 78, IQR 70–84), and physicians (median 85, IQR 78–92) displayed moderate median scores but wide interquartile ranges.

#### 3.2.2. Attitudes

**Figure 2:**
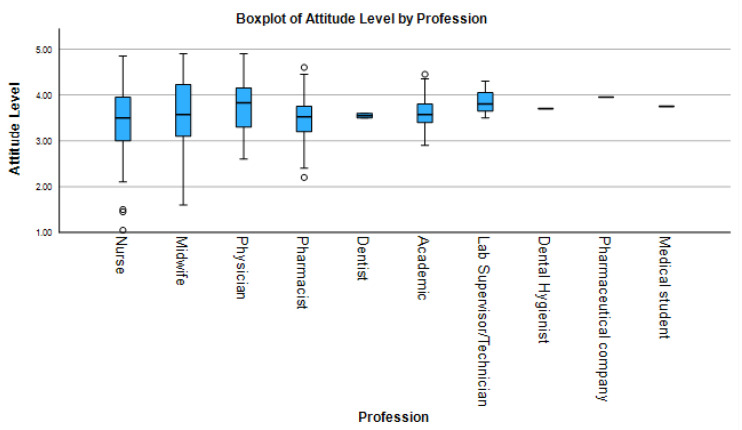
Boxplot comparisons of HPV-related attitude scores across healthcare professions. Center lines show medians, boxes represent interquartile ranges (IQR), and whiskers extend to 1.5× IQR. Individual points denote outliers. Brackets show significant Dunn’s test comparisons with effect sizes.

Attitudes toward HPV vaccination were generally positive across all professional groups (Figure 2), though significant variation existed. Medical students (median 4.5, IQR 4.3–4.8) and pharmaceutical professionals (median 3.7, IQR 3.3–4.0) reported the highest median attitude scores, with narrow interquartile ranges suggesting strong consensus. In contrast, nurses (median 3.8, IQR 3.4–4.1) and pharmacists (median 3.7, IQR 3.3–4.0) exhibited more variable responses, with lower outliers suggesting divergent perceptions. Physicians (median 4.2, IQR 3.8–4.5) and midwives (median 3.9, IQR 3.6–4.3) also displayed broader score distributions.

#### 3.2.3. Practices

**Figure 3:**
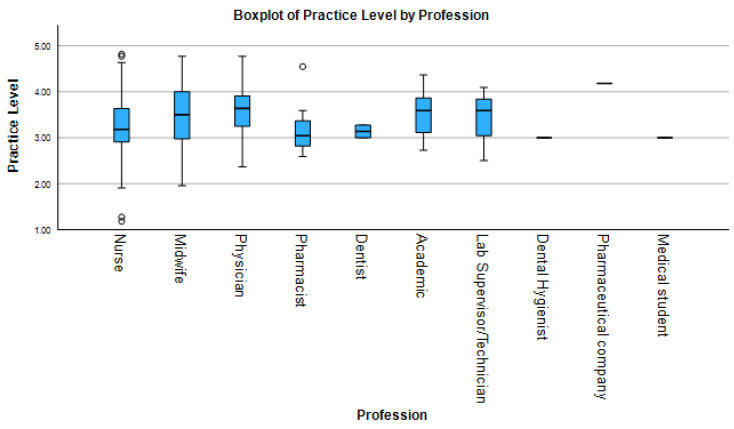
Boxplot comparisons of HPV-related practice scores across healthcare professions. Center lines show medians, boxes represent interquartile ranges (IQR), and whiskers extend to 1.5× IQR. Individual points denote outliers. Brackets show significant Dunn’s test comparisons with effect sizes.

HPV-related clinical practice scores varied significantly across professional groups (Figure 3). Academics (median 4.3, IQR 4.0–4.6), physicians (median 4.5, IQR 4.1–4.8), and midwives (median 4.2, IQR 3.9–4.5) reported the highest levels of clinical engagement, with median scores above 4.2. Pharmacists (median 3.7, IQR 3.3–4.0) also showed relatively high scores, but with no observed variability, suggesting adherence to standardized protocols. Conversely, medical students (median 3.8, IQR 3.5–4.1) showed lower and less variable practice scores, likely reflecting limited clinical responsibilities. Data for laboratory technicians and dental hygienists are not provided in the table. Nurses (median 3.9, IQR 3.5–4.2) exhibited the widest range of practice scores.

The results of descriptive analysis, inferential statistics, and post hoc comparisons are summarized in Table 2. Kruskal–Wallis tests confirmed statistically significant differences across professions in all KAP domains: knowledge (H = 28.7, *p* < 0.001, ε^2^ = 0.18), attitudes (H = 12.4, *p* = 0.015, ε^2^ = 0.08), and practices (H = 34.2, *p* < 0.001, ε^2^ = 0.21). Post hoc Dunn’s tests revealed that physicians scored significantly higher in knowledge compared to nurses (*p* < 0.001, r = 0.42) and pharmacists (*P* = 0.018, r = 0.35); in attitudes compared to pharmacists (*p* = 0.018); and in practice compared to both nurses (*p* < 0.001, r = 0.45) and pharmacists (*p* = 0.031, r = 0.33). Midwives outperformed nurses in practice (*p* = 0.002, r = 0.37).

### 3.3. Correlational Analyses

**Figure 4:**
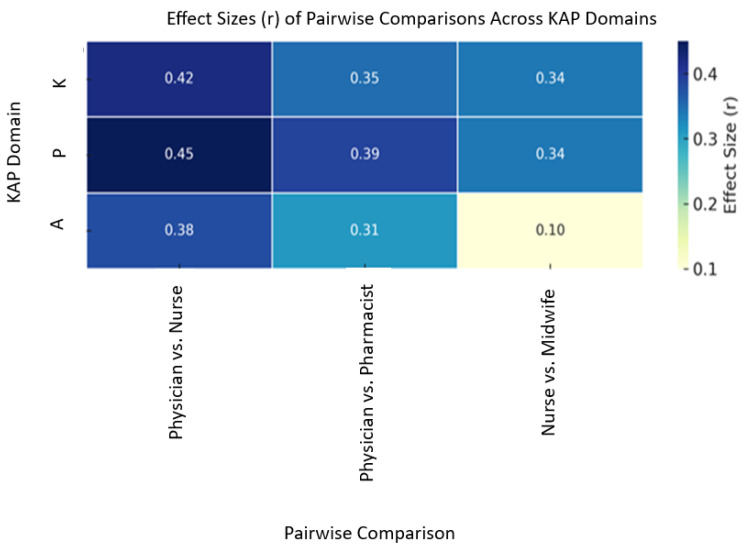
Heatmap of rank-biserial correlations from Dunn’s post hoc tests. Blue/yellow indicate directionality of significant differences (*p* <0.001).

Correlation analyses among the continuous variables revealed a weak but statistically significant negative association between knowledge scores and both attitude (r = −0.25, *p* < 0.001) and practice scores (r = −0.22, *p* < 0.001). In contrast, a strong positive correlation was observed between attitude and practice scores (r = 0.72, *p* < 0.001) (Figure 4).

### 3.4. Recommendation Behavior and Associated Predictors

Among participants, 64.5% reported recommending the HPV vaccine. Firth’s penalized logistic regression identified three significant predictors (Table 3). Knowledge scores were inversely associated with recommendation behavior (adjusted odds ratio [aOR] = 0.44, 95% CI: 0.20–0.93, *p* = 0.032). In contrast, both attitudes (aOR = 3.89, 95% CI: 1.84–8.61, *p* < 0.001) and practices (aOR = 5.02, 95% CI: 2.19–12.51, *p* < 0.001) were positively associated with vaccine recommendation. Institutional affiliation (public vs. private), geographic location (urban vs. rural), and professional category were initially significant in univariate analyses but did not remain significant in the multivariate model.

Prior HPV vaccination training showed no significant association with profession (*p* > 0.05). Similarly, gender did not significantly influence vaccine recommendation behavior (*p* = 0.845), despite a significant difference in years of formal education between male and female professionals (*p* = 0.005).

### 3.5. Scale Reliability

As presented in Table 4, the KAP questionnaire demonstrated satisfactory internal consistency. Cronbach’s alpha values were 0.87 for knowledge, 0.83 for attitudes, and 0.89 for practices, supporting the internal reliability of all domains for inferential analysis.

## 4. Discussion

This study examined the influence of knowledge, attitudes, and practices (KAP) on healthcare professionals’ engagement with human papillomavirus (HPV) vaccination in Amman, Jordan. Our findings revealed that while attitudes and practices toward HPV vaccination were generally more favorable than knowledge levels, significant inter-professional variation existed across all three KAP domains. Notably, attitudes and clinical practices emerged as the strongest independent predictors of HPV vaccine recommendation behavior, underscoring their pivotal role in shaping provider-driven public health outcomes. This highlights the importance of considering not only factual knowledge but also the affective and behavioral dimensions when designing interventions to improve HPV vaccination rates.

The demographic composition of our sample revealed role-based differences consistent with global trends. For example, studies in New York [27] have shown that physicians and dentists are more likely than pharmacists to recommend the HPV vaccine, likely due to their greater clinical experience and higher patient volume. Similarly, research in Saudi Arabia [28] demonstrated that physicians exhibited stronger recommendation practices than nurses or technicians, particularly when structured training was provided. Conversely, studies in Serbia [21] highlighted persistent knowledge gaps among medical students and technicians, emphasizing the need for early curricular integration of HPV vaccination education. In Southern China [29], community-based providers showed higher vaccine engagement than hospital staff, possibly reflecting their role in primary prevention. Furthermore, research in Kazakhstan [30] indicated that provider role and institutional context, rather than gender, were the primary predictors of recommendation behavior.

In our Amman-based study, although male participants demonstrated higher educational attainment, gender did not significantly influence HPV vaccine recommendation behavior. This aligns with findings from Saudi Arabia [28] and Kazakhstan [30], which emphasized that professional role and institutional factors are stronger predictors of vaccine advocacy than gender. This suggests that targeted interventions should focus on addressing professional-specific knowledge gaps and fostering supportive attitudes and practices within different healthcare settings.

Expanding on the interprofessional variations observed, our study revealed notable differences in HPV-related knowledge, attitudes, and practices (KAP) across professional groups. The observed disparities in vaccine recommendation behavior are not solely explained by differences in knowledge levels, as previously discussed. For ex-ample, medical students, pharmacists, and dental hygienists demonstrated stronger attitudinal scores toward HPV vaccination. This aligns with findings from Serbia [21], where medical students expressed more favorable views toward vaccination than their nursing and technical counterparts. Similarly, studies in Jordan [14] and Saudi Arabia [28] indicated that physicians exhibited higher practice scores than nurses and technicians, a difference likely attributable to variations in curricular exposure and clinical authority.

These interprofessional disparities underscore the need for targeted interventions. Inconsistent training access and role-specific responsibilities likely contribute to these variations. Research from Southern China [29] supports this, showing that community health workers, due to their involvement in grassroots preventive services, demonstrated greater HPV vaccine engagement than hospital-based providers. Our findings resonate with the World Health Organization’s recommendations [31] for standardized training protocols and inclusive policy frameworks to ensure equitable vaccine promotion across all healthcare settings. Therefore, future educational interventions should be tailored to address the specific needs and contexts of different professional groups, considering factors such as training access, role-specific responsibilities, and the need to foster positive attitudes and confident clinical practices.

Correlational analyses further illuminated the complex interplay between KAP and HPV vaccine recommendation behavior. A strong positive correlation (r = 0.72, *p* < 0.001) emerged between attitude and practice, confirming that positive attitudes are key behavioral drivers in vaccine advocacy and health promotion, consistent with global literature [24,25]. This reinforces the earlier observation that fostering positive attitudes is crucial for increasing HPV vaccine uptake.

Conversely, a weak inverse correlation between knowledge and practice under-scored the well-documented knowledge–behavior paradox. This paradox, where in-formation alone does not guarantee clinical action, is supported by a systematic review by Vincent et al. [32] which found that while healthcare professionals in the MENA region often possessed moderate knowledge about HPV, translating this knowledge into recommendation behavior remained challenging, particularly in the face of vaccine safety concerns and weak institutional support.

This knowledge–practice disconnect may also stem from analytic hesitation or a fear of patient mistrust, as highlighted in recent global research. Kassymbekova et al. [30] emphasized that provider role and institutional culture are stronger predictors of vaccine uptake advocacy than knowledge alone, especially in contexts with ambiguous guidelines or inconsistent messaging. These findings collectively suggest that while knowledge forms a foundation, emotional, contextual, and organizational factors significantly influence the translation of knowledge into clinical practice. Therefore, interventions must address not only knowledge gaps but also the broader socio-cultural and institutional barriers that hinder the adoption of evidence-based practices.

Our study revealed an HPV vaccine recommendation rate of 64.5% among healthcare professionals in Amman, Jordan. While this rate surpasses previous reports from Jordan, it remains lower than rates observed in countries with established immunization programs, such as the United States and the United Kingdom [5,7]. This disparity underscores the need for targeted interventions to improve vaccine uptake in Jordan.

Consistent with findings from Egypt, Pakistan, and Saudi Arabia [33,34,35], our multivariate analysis indicated that favorable attitudes and confident clinical practices, rather than knowledge alone, were the strongest predictors of vaccine recommendation. This observation reinforces the “knowledge-behavior paradox,” where possessing knowledge does not automatically translate into action. Even after controlling for profession and demographics, knowledge demonstrated a weak or even negative association with vaccine recommendation in our study.

This paradox is supported by international research. Studies in the United States [36] have shown that cognitive overload and risk sensitivity can lead to professional hesitation, despite high levels of factual understanding. A global review [37] identified institutional ambiguity, inadequate training in patient communication, and an over-emphasis on risk as significant barriers to vaccine promotion. Furthermore, research in Iran [38] highlighted the importance of trust in institutional systems in shaping provider attitudes, exceeding the influence of factual knowledge. Similarly, studies in the United States [36] demonstrated that healthcare professionals serving vulnerable communities prioritized relational trust, cultural alignment, and effective communication strategies over biomedical expertise when recommending vaccination. These cross-national findings strongly suggest that effective HPV vaccine promotion requires a multi-faceted approach that extends beyond simply in-creasing knowledge. Strengthening communication skills, building institutional trust, and promoting confident recommendation behaviors among healthcare professionals are crucial for improving HPV vaccine uptake.

The lack of a significant association between prior HPV training and professional role in our study mirrors regional trends observed in Jordan and neighboring countries [14,15,34], where training programs are often fragmented, inconsistent, and optional. Although our analysis did not reveal a statistically significant association, HPV-focused training may still indirectly improve provider confidence and enhance behavioral consistency [12,20]. However, studies from Egypt and Pakistan [33,34] highlight that training alone is insufficient to guarantee consistent vaccine advocacy. Effective training must be coupled with clear institutional protocols and incorporate communication and confidence-building components. The World Health Organization (WHO) emphasizes integrating such training into Continuing Professional Development (CPD) systems and licensure renewal pathways to ensure sustainable integration and equitable access [1,31].

This need for comprehensive, context-specific training is further underscored by research from Saudi Arabia [35], where even structured training programs failed to fully address knowledge gaps and improve recommendation practices unless complemented by institutional incentives and supervisory support. In the United States [20], unclear public health messaging during vaccine rollouts hindered uptake, even among trained professionals. Qualitative research among African American communities in Tennessee [36] further emphasizes the need for training that addresses both technical knowledge and cultural communication challenges to effectively improve real-world vaccination rates. Therefore, successful HPV vaccination promotion requires a holistic approach that combines effective training with supportive institutional structures and clear, culturally sensitive communication strategies.

## 5. Strengths and Limitations

This study presents several methodological strengths. The sample size was adequate for the planned statistical analyses, and the high response rate enhances internal validity. Inclusion of multiple healthcare professional categories enabled comprehensive interprofessional comparisons, offering valuable insight into the heterogeneity of HPV-related knowledge, attitudes, and practices.

Nonetheless, important limitations must be acknowledged. First, the 60.8% response rate (304/500 completed questionnaires) may affect the generalizability of the findings, despite the sample size exceeding the calculated minimum for statistical analysis. The use of multiple distribution methods (institutional email, professional networks, and social media) hinders precise determination of the total eligible population exposed to the survey, potentially introducing self-selection bias. Participants may not fully represent all healthcare professionals in Amman, Jordan. Furthermore, the convenience sample from selected Amman institutions may not reflect the characteristics and professional distributions in other regions. Finally, reliance on self-reported data introduces potential response bias, including social desirability effects and recall inaccuracies, potentially leading to overestimation of favorable attitudes and practices.

The cross-sectional design, although effective in capturing diverse views at a single point in time, precludes causal inference and hinders tracking of behavioral change over time. The lack of follow-up on actual recommendation or vaccination behaviors further limits the ability to evaluate the translation of reported attitudes into clinical action. Moreover, the study period coincided with ongoing public health campaigns and media coverage related to HPV vaccination in the region, which may have influenced awareness levels and response patterns—potentially amplifying certain knowledge or attitudinal trends beyond typical baselines.

Despite these constraints, the study employed robust psychometric validation, appropriate statistical modeling, and subgroup analyses, supporting the credibility and contextual relevance of its findings.

## 6. Conclusions

This study revealed moderate HPV vaccine recommendation rates among Jordanian healthcare professionals, with significant variation across professions. Attitudes and clinical practices, rather than knowledge levels, proved to be the strongest predictors of recommendations. This “knowledge-behavior paradox” underscores the need for interventions prioritizing the practical application and effective framing of information, not simply increased knowledge. Addressing these profession-specific disparities requires tailored educational programs extending beyond knowledge dissemination, incorporating role-specific case studies, simulation-based learning, and peer-led discussions to build confidence and facilitate real-world application. Effective communication strategies to address vaccine hesitancy are also crucial. Finally, stronger institutional support, including integrating HPV training into CPD frameworks and linking it to licensure, is essential for consistent practice.

## Figures and Tables

**Table 1:** The socio-demographic characteristics of healthcare professionals at various hospitals of Amman (*n* = 304).

Characteristics		Frequency	Percent (%)
Age (years old)	18–29	92	30.3
	30–39	98	32.2
	40–49	92	30.3
	50–59	18	5.9
	60 or greater	4	1.3
Gender	Male	63	20.7
	Female	241	79.3
Religion	Muslim	291	95.7
	Christian	13	4.3
Profession	Nurse	134	44.1
	Midwife	68	22.4
	Physician	52	17.1
	Pharmacist	30	9.9
	Dentist	2	0.7
	Academic	12	3.9
	Lab Supervisor/Technician	3	1.0
	Dental Hygienist	1	0.3
	Pharmaceutical company	1	0.3
	Medical student	1	0.3
Years of Education	0–5	171	56.3
	6–10	93	30.6
	11 or more	40	13.2
Working Setting	Rural	61	20.1
	Urban	243	79.9
Institution Type	Private	103	33.9
	Public	192	63.2
	Military	9	3.0

**Table 2:** Comparison of HPV-Related Knowledge, Attitudes, and Practices Across Professions (Kruskal–Wallis Test Results, *n* = 304).

Profession	*n*	Knowledge	Attitude	Practice	H	*p*	ε^2^	Post Hoc	Adj. *p*	r
		Median (IQR)	Median (IQR)	Median (IQR)	(df = 5)					
Physician	30	85 (78–92)	4.2 (3.8–4.5)	4.5 (4.1–4.8)	28.7	<0.001	0.18	vs. Nurse, Pharmacist (K)	<0.001, 0.018	0.42, 0.35
Nurse	60	72 (65–80)	3.8 (3.4–4.1)	3.9 (3.5–4.2)	12.4	0.015	0.08	vs. Physician, Midwife (P)	<0.001, 0.002	0.45, 0.37
Midwife	42	78 (70–84)	3.9 (3.6–4.3)	4.2 (3.9–4.5)				vs. Nurse (P)	0.002	0.37
Pharmacist	28	70 (62–76)	3.7 (3.3–4.0)	3.7 (3.3–4.0)	34.2	<0.001	0.21	vs. Physician, Academic (P)	0.018, 0.031	0.35, 0.33
Academic	48	80 (75–88)	4.0 (3.7–4.4)	4.3 (4.0–4.6)				vs. Pharmacist (P)	0.031	0.33
Medical Student	96	88 (80–94)	4.5 (4.3–4.8)	3.8 (3.5–4.1)						

Note: H = Kruskal–Wallis test statistic; ε^2^ = Epsilon-squared effect size (interpretation: 0.01 = small, 0.06 = medium, 0.14 = large); Adjusted *p*-values via Dunn–Bonferroni post hoc test; *p* = Practice domain, K = Knowledge domain, P = Practices domain, r = Effect size.

**Table 3:** Predictors associated with recommendation of HPV vaccine (*n* = 304).

Predictor		Frequency (%)	Uni-Variate Analysis		Firth Penalized Logistic Regression	
			*χ* ^2^	*p*	*aOR* (95% *CI*)	*p*
**Overall**		196 (64.5%)	-	-		
**Age**			4.71	0.318		
	18–29	59.3 (30.1)				
	30–39	63.2 (31.1)				
	40–49	59.3 (32.1)				
	50–59	11.6 (4.6)				
	60≤	2.6 (2.0)				
**Gender**			0.59	0.439		
	Male	40.6 (19.4)				
	Female	155.4 (80.6)				
**Profession**			20.49	**0.015**		
	Nurse	86.4 (38.3)			1 (Ref)	
	Midwife	43.8 (22.4)			0.86 (0.34, 2.21)	0.754
	Physician	33.5 (22.4)			2.14 (0.66, 7.50)	0.209
	Pharmacist	19.3 (8.7)			1.28 (0.48, 3.49)	0.623
	Dentist	1.3 (1.0)			4.82 (0.31, 718.21)	0.283
	Academic	7.7 (4.6)			1.72 (0.36, 9.47)	0.501
	Lab Supervisor/Tech	1.9 (1.5)			2.19 (0.12, 344.66)	0.630
	Dental Hygienist	0.6 (0.5)			1.66 (0.08, 259.49)	0.759
	Pharmaceutical company	0.6 (0.5)			0.20 (0.01, 33.04)	0.421
	Medical student	0.6 (0.0)			0.29 (0.00, 6.93)	0.450
**Years of Education**			5.60	0.61		
	0–5	110.3 (52.6)				
	6–10	60.6 (31.1)				
	11≤	25.8 (16.3)				
**Working Setting**			7.79	**0.005**		
	Rural	39.3 (15.3)			1 (Ref)	
	Urban	156.7 (84.7)			1.07 (0.53, 2.15)	0.8602
**Institution Type**			7.24	**0.027**		
	Private	66.4 (38.8)			1 (Ref)	
	Public	123.8 (57.7)			0.78 (0.39, 1.55)	0.4733
	Military	5.8 (3.6)			0.35 (0.05, 3.05)	0.3143
**HPV Vaccine Training**			0.85	0.355		
	Yes	16.1 (7.1)				
	No	179.9 (92.9)				
**Knowledge Level**		196 (64.5)	36.52	**0.013**	0.44 (0.20, 0.93)	**0.032**
**Attitude Level**		196 (64.5)	120.63	**<0.001**	3.89 (1.84, 8.61)	**<0.001**
**Practice Level**		196 (64.5)	115.76	**<0.001**	5.02 (2.19, 12.51)	**<0.001**

*Note:* Abbreviations: *aOR* = adjusted odds ratio; *CI* = confidence interval; Ref = reference category. Firth’s penalized regression addressed quasi-complete separation. Bolded *p*-values indicate significance (*p* < 0.05). Univariate χ^2^ tests were used for categorical variables. Effect estimates for age, gender, and profession were adjusted for knowledge, attitude, and practice scores.

**Table 4:** Reliability Statistics for Knowledge, Attitude, and Practice Scales.

Scale	Cronbach’s Alpha	Number of Items
Knowledge	0.761	10
Attitude	0.854	9
Practice	0.870	23

## Data Availability

The data supporting the findings of this study are available within the article and its Supplementary Materials.

## Supplementary Materials

The following supporting information can be downloaded at: https://www.mdpi.com/article/10.3390/idr17050126/s1, Supplementary S1: Questionnaire: “Preliminary Survey Questionnaire”.
