# Supplementary material for: Knowledge, Attitudes, and Practices Associated with Human Papillomavirus Vaccine Recommendation Among Healthcare Professionals: A Cross-Sectional Study
Source: Infect Dis Rep. 2025 Oct 9;17(5):126. doi: 10.3390/idr17050126 (PMC12564430; doi:10.3390/idr17050126)
Supplement: Supplementary file 1 [file idr-17-00126-s001.zip › idr-3776558-supplementary.pdf]

Preliminary Survey Questionnaire

Instructions: Please circle your responses to the questions, some questions will also require you to write in your response. There is no right or wrong answers. Please tell us what you really think.

Demographics:

1. What is your age in years?
   (a) 18-29 (b) 30-39 (c) 40-49 (d) 50-59 (e) 60 or greater
2. What is your gender?
   (a) Male (b) Female
3. Where do you live?
   (a) Rural (b) Urban (e.g. city)
4. Do you have children?
   (a) No (b) Yes 4A. If yes, how many are female ______, how many male_____?
5. If comfortable disclosing, what religion do you practice?
   (a) Christian (b) Muslim (c) Other (list):___________________

Education/training/experience

6. What is your profession?
   (a) Physician (b) Pharmacist (c) Nurse (d) Other health worker (list): ____________
7. How many years of education have you had after primary school (high school)? _____________
8. In what setting do you work?
   (a) Rural (b) Urban (e.g. city)
9. What type of institution do you work for?
   (a) Private (b) Public (e.g. government) (c) Other (list):__________________
10. Have you received training or certification for vaccine administration?
    (a) No (b) Yes
    A. If yes, what was the training or certification?
       ____
    B. If yes, how many years have you been certified? _________________
    C. If yes, did the training include HPV vaccine (human?
       (a) No (b) Yes
11. Have you ever administered or recommended the HPV vaccine (human papillomavirus)?
    (a) No (b) Yes → approximately how many times? _____________________
    A. If no, are you familiar with HPV vaccine?
       (a) No (b) Yes
12. Approximately how many patients do you see monthly? _____________________
    A. What percent are female? ___________________
    B. What percent of females are ages 9-26 years old? _____________
13. What sources of information do you use for information about HPV/cervical cancer or other medical information (circle all that apply)?
    A. Colleagues
    B. Journals
    C. Government
    D. Other (list): ______________________

Background knowledge

14. Have you ever heard of cervical cancer?
    (a) No (b) Yes (c) Unsure
15. A Pap smear test or Pap test is a screening tool that is used to check for problems in the cervix. Have you ever administered a Pap smear test?
    (a) No (b) Yes (c) Unsure
16. There is no known way to prevent cervical cancer.
    (a) False (b) True (c) Unsure
17. Have you ever heard of Human Papillomavirus (HPV) infection?
    (a) No (b) Yes (c) Unsure
18. HPV (human papillomavirus) is a sexually transmitted disease/infection.
    (a) False (b) True (c) Unsure
19. There is no cure for HPV (human papillomavirus) but it can be controlled.
    (a) False (b) True (c) Unsure
20. Having HPV (human papillomavirus) puts one at risk for cervical cancer.
    (a) False (b) True (c) Unsure
21. HPV (human papillomavirus) is an infection that only affects women.
    (a) False (b) True (c) Unsure
22. The HPV (human papillomavirus) vaccine is available in Nigeria.
    (a) False (b) True (c) Unsure
23. Only one dose of the HPV vaccine is required for optimal protection.
    (a) False (b) True (c) Unsure

Provider Perception and Practice

24. Rate the severity of disease for HPV (human papillomavirus) infection:
    (1) Not severe (2) Slightly severe (3) Somewhat severe (4) Very severe (5) Extremely severe
25. Rate the severity of disease for cervical cancer:
    (1) Not severe (2) Slightly severe (3) Somewhat severe (4) Very severe (5) Extremely severe
26. When deciding to administer or recommend HPV vaccination which of the following are important factors (circle a number on the scale):

A.	Preventing cervical cancer	Not important 1 2 3 4 5 Extremely important
B.	Vaccine availability	Not important 1 2 3 4 5 Extremely important
C.	Vaccine effectiveness	Not important 1 2 3 4 5 Extremely important
D.	Vaccine safety	Not important 1 2 3 4 5 Extremely important
E.	Cost of vaccine	Not important 1 2 3 4 5 Extremely important
F.	Patient accepting vaccine	Not important 1 2 3 4 5 Extremely important
G.	Government recommendation	Not important 1 2 3 4 5 Extremely important
H.	Other providers recommendation	Not important 1 2 3 4 5 Extremely important

27. Which of the factors listed above (#26) is MOST important: ___________________________

USE THE KEY IN THE BOX TO ANSWER THE FOLLOWING QUESTIONS

28. Vaccines prevent disease
    (1) SD (2) D (3) N (4) A (5) SA
29. Generally, I recommend vaccines to patients who meet the eligibility criteria
    (1) SD (2) D (3) N (4) A (5) SA
30. HPV vaccine prevents cervical cancer
    (1) SD (2) D (3) N (4) A (5) SA
31. I recommend the HPV vaccine to patients who are eligible
    (1) SD (2) D (3) N (4) A (5) SA

Key:
(1) SD-Strongly disagree
(2) D-Disagree
(3) N-Neutral/neither
(4) A- Agree
(5) SA- Strongly agree

32. I know who is eligible for the HPV vaccine
    (1) SD (2) D (3) N (4) A (5) SA
33. I have access to the HPV vaccine
    (1) SD (2) D (3) N (4) A (5) SA
34. My patients are concerned about their risk for cervical cancer
    (1) SD (2) D (3) N (4) A (5) SA
35. My patients are at risk for acquiring HPV infection
    (1) SD (2) D (3) N (4) A (5) SA
36. My patients are at risk for acquiring cervical cancer
    (1) SD (2) D (3) N (4) A (5) SA
37. The benefits of HPV vaccination outweigh the risks
    (1) SD (2) D (3) N (4) A (5) SA

Key:
(1) SD-Strongly disagree
(2) D-Disagree
(3) N-Neutral/neither
(4) A- Agree
(5) SA- Strongly agree

Each of the following questions refer to HPV vaccine administration to eligible patients

38. If I administer the HPV vaccine, I will feel that I am doing something positive for the patient
    (1) SD (2) D (3) N (4) A (5) SA
39. It causes a lot of worry and concern for a patient if they do not receive the HPV vaccination
    (1) SD (2) D (3) N (4) A (5) SA
40. If I administer the HPV vaccine, I will help prevent cervical cancer
    (1) SD (2) D (3) N (4) A (5) SA
41. For the patient, preventing problems with cervical cancer is desirable
    (1) SD (2) D (3) N (4) A (5) SA
42. Offering and administering the HPV vaccine is harmful
    (1) SD (2) D (3) N (4) A (5) SA
43. Patients eligible for the vaccine would approve my offering the HPV vaccination
    (1) SD (2) D (3) N (4) A (5) SA
44. The approval of my patients is important to me
    (1) SD (2) D (3) N (4) A (5) SA
45. Other health workers offer the HPV vaccination
    (1) No (2) Yes (3) Maybe (4) Unsure
46. Health workers **should** offer the HPV vaccination
    (1) No (2) Yes (3) Maybe (4) Unsure
47. The government would approve my offering the HPV vaccination
    (1) No (2) Yes (3) Maybe (4) Unsure
48. The government's approval of my clinical practice is important to me
    (1) SD (2) D (3) N (4) A (5) SA
49. I am likely to offer and administer the HPV vaccine if the **cost is affordable**
    (1) SD (2) D (3) N (4) A (5) SA
50. I am likely to offer and administer the HPV vaccine if the **vaccine is more accessible**
    (1) SD (2) D (3) N (4) A (5) SA
51. I am likely to offer/administer the HPV vaccine if it is **more acceptable** by my community
    (1) SD (2) D (3) N (4) A (5) SA
52. People who are important to me think I should NOT offer and administer the HPV vaccine
    (1) SD (2) D (3) N (4) A (5) SA
53. I **intend** to offer/administer the HPV vaccine to **all** of my eligible patients
    (1) SD (2) D (3) N (4) A (5) SA

Key:
(1) SD-Strongly disagree
(2) D-Disagree
(3) N-Neutral/neither
(4) A- Agree
(5) SA- Strongly agree

54. I feel under social pressure to offer/administer the HPV vaccine
    (1) SD (2) D (3) N (4) A (5) SA
55. I am confident that I can offer/administer the HPV vaccine if I want to
    (1) SD (2) D (3) N (4) A (5) SA
56. Whether I offer/administer the HPV vaccine is entirely up to me
    (1) SD (2) D (3) N (4) A (5) SA
57. It is **easy** for me to offer/administer the HPV vaccine to my patients
    (1) SD (2) D (3) N (4) A (5) SA
58. I **want** to offer/administer the HPV vaccine to each of my eligible patients
    (1) SD (2) D (3) N (4) A (5) SA
59. It is expected of me that I offer/administer the HPV vaccine to eligible patients
    (1) SD (2) D (3) N (4) A (5) SA
60. Out of the next 10 patients you see who are eligible for the HPV vaccine, for how many would you expect to offer/administer the HPV vaccine?
    (1) None, 0% (2) Some, 1-59% (3) Most, 50-90% (4) All, 90-100%
